# Risk factors for bronchiectasis in patients with chronic obstructive pulmonary disease: a systematic review and meta-analysis

**DOI:** 10.6061/clinics/2021/e2420

**Published:** 2021-04-07

**Authors:** XinXin Zhang, LiJian Pang, XiaoDong Lv, HaoYang Zhang

**Affiliations:** I Liaoning University of Traditional Chinese Medicine, Shenyang, China; II Liaoning University of Traditional Chinese Medicine Affiliated Hospital, Shenyang, China

**Keywords:** Chronic Obstructive Pulmonary Disease, Bronchiectasis, Risk Factors, Meta-Analysis

## Abstract

The risk factors of bronchiectasis in patients with chronic obstructive pulmonary disease have not yet been established. This systematic review and meta-analysis aimed to investigate and identify potential risk factors for patients with chronic obstructive pulmonary disease accompanied by bronchiectasis. We reviewed eight electronic journal databases from their inception to November 2019 for observational studies with no language restrictions. The Newcastle-Ottawa Scale was applied to evaluate the quality of the literature. Binary variables were pooled using odds ratios and continuous variables using the standardized mean difference with 95% confidence intervals. The confidence of evidence was assessed according to the grading of the recommendations assessment, development, and evaluation method. Eight case-control studies met the inclusion criteria. Tuberculosis history, smoking history, hospitalization stays, admissions in the past year, and duration of symptoms were considered risk factors. In addition, the ratio between the forced expiratory volume in 1s and forced vital capacity, the percentage of forced expiratory volume in 1s, the forced expiratory volume in 1s as a percentage of the predicted value, purulent sputum, purulent mucus sputum, positive sputum culture, *Pseudomonas aeruginosa* infection, arterial oxygen pressure, daily dyspnea, C-reactive protein, leukocytes, and the percentage of neutrophils were found to be closely related to bronchiectasis. However, these were not considered risk factors. The evidence of all outcomes was judged as “low” or “very low.” Additional prospective studies are required to elucidate the underlying risk factors and identify effective preventive interventions.

## INTRODUCTION

Chronic obstructive pulmonary disease (COPD) is one of the main causes of global morbidity and mortality (1) and is characterized by partially reversible, persistent airflow limitation associated with chronic airway inflammation and emphysema (2). COPD is a complex heterogeneous disease (3). The clinical presentation and structural abnormalities of the lung can vary greatly between patients (3). With the increasing application of computed tomography (CT) in the evaluation of patients with COPD, previously unrecognized bronchiectasis is being identified (4). Ko et al. (33) defined the most accepted diagnostic criteria for bronchiectasis. Bronchiectasis is characterized by the irreversible widening of medium to small-sized airways, inflammation, chronic bacterial infection, and destruction of the bronchial walls (5).

Some studies have pointed out that bronchiectasis and COPD may co-exist as an overlap syndrome (6). Bronchiectasis was first defined as a comorbidity of COPD in the Global Chronic Obstructive Pulmonary Disease Initiative 2014 guidelines (7). This change was retained in the 2015 updated version and emphasized the impact of bronchiectasis on the natural history of COPD (6). Multiple studies have shown that bronchiectasis in patients with COPD is associated with increased bronchial inflammation, frequent colonization of potentially pathogenic microorganisms, and severe airflow obstruction (8). Bronchiectasis tends to adversely affect the clinical status of patients with COPD, lower their exercise capacity and quality of life, seriously influence the state of psychology, and cause a poor prognosis (9). Moreover, some cases may be obliged to adopt more efficient and sustained antibiotic therapy, and inhaled corticosteroids may not be suitable for patients with bacterial colonization or recurrent lower respiratory infections (10).

Therefore, identifying the potential risk factors for bronchiectasis in patients with COPD could lead to earlier detection and diagnosis, better guidance for management, more effective treatments, and improvement of health status. However, the risk factors for bronchiectasis in patients with COPD have not been fully confirmed. Several observational studies have investigated them but with small sample sizes. In addition, some contradictory results were found in these studies. For example, Arram and Elrakhawy (11) found that age is a potential risk factor, but the studies by Martínez-García et al. (8) and Yu et al. (12) did not support this result. Thus, this systematic review and meta-analysis aimed to summarize the current evidence of observational studies and then investigate and identify potential risk factors for bronchiectasis in patients with COPD.

## MATERIALS AND METHODS

### Research registration

This study was registered on the International Prospective Register of Systematic Reviews (PROSPERO no. CRD 42020171581) and was carried out according to the Meta-analysis Reporting Guide for Observational Research (13).

### Search strategy

We conducted a comprehensive retrieval of eight electronic journal databases, including PubMed, Cochrane Library, Embase, Web of Science, China National Knowledge Infrastructure, Chinese Biomedical Literature Database, WanFang Database, and Chinese Scientific Journal Database. We reviewed these databases from their inception to November 2019 for observational studies with no restrictions placed on the language of publications. In addition, the bibliographies of identified articles and grey literature were also searched to avoid any omissions. The search strategy of the PubMed database is shown in Table 1, and we adjusted it according to the characteristics of others.

### Eligibility criteria

The inclusion criteria were as follows: 1) eligible observational studies were identified if the risk factors for bronchiectasis in COPD were demonstrated; 2) diagnosis of COPD complies with any version of reliable and accepted guidelines with clear diagnostic criteria and bronchiectasis diagnosed by objective imaging methods such as high-resolution CT (HRCT), CT scan, or chest X-ray (14); 3) studies with all study participants older than 18 years; 4) studies comparing patients with COPD and bronchiectasis in the research group to patients with COPD without bronchiectasis in the control group to identify risk factors; and 5) studies with complete experimental data and results.

The exclusion criteria were as follows: 1) duplicate articles, 2) case report, 3) letters, 4) meeting abstracts, 5) animal experiments, 6) review articles, 7) comment articles, 8) low quality studies, and 9) studies with incomplete data and unclear outcomes.

### Literature screening

All retrieved studies were imported into the Note Express 3.2.0.7350 software (Beijing Aegean Music Technology Co., Ltd.) to delete any duplicates. Two researchers (Zhang XX, Zhang HY) independently screened the titles and abstracts against the established inclusion and exclusion criteria and then downloaded the remaining studies for further screening by reading the full text. If any disagreements occurred, a consensus was reached through discussion or adjudication by a third senior researcher (Pang LJ).

### Data extraction

The key characteristics of the included articles were extracted independently by two reviewers (Zhang XX, Zhang HY) using a predefined form. The following data items were collected from each study: the first author, publication year, primary locality of the study, sample size (research group/control group), outcomes, range of age (research group/control group), sex distribution (male/female), diagnostic criteria, and funding. If any important information elements were missing, we attempted to contact the authors for the desired data. If any disagreements occurred during this process, the two reviewers reached a consensus through consultation or adjudication by a third senior investigator (Pang LJ).

### Quality assessment

Two researchers (Zhang XX, Zhang HY) independently and separately applied the Newcastle-Ottawa Scale (NOS) (15) to evaluate the quality of the included literature, which contains three aspects: selection, comparability, and exposure/outcome. Those studies with a score of 5 or more were classified as high quality, while those with a score lower than 5 were classified as low quality (16). To ensure the reliability of the results, low quality literature were not be included in the meta-analysis. Any disagreement during this period was discussed with a third senior researcher (Lv XD). The AMSTAR 2 checklist was used to evaluate the methodological quality of this meta-analysis by two researchers independently (Zhang XX, Zhang HY). This checklist includes 16 criteria. The methodological quality score ranged from 0 to 16. Scores of 15-16, 12-14, 9-11, 6-8, and 3-5 items were evaluated as excellent, very good, good, acceptable, and deficient, respectively (17). Disagreements were resolved by consensus with a third investigator (Lv XD).

### Statistical analysis

The Stata13.1 software (Stata-Corp LP, College Station TX77845) was used for the meta-analysis. The Q-test and I^2^ values were applied to measure the inter-study heterogeneity. When the *p*-value of Q-test>0.1 and *I* ^2^<50%, a fixed-effects model was applied; otherwise, a random-effects model was used. Binary variables were expressed using the odds ratio with 95% confidence interval (CI) and continuous variables by the standardized mean difference with 95% CI. Forest plots were created using GraphPad Prism version 7.00 software. A subgroup analysis was used to explore the potential confounding factors for significant heterogeneity, such as age, country, literature quality, and publication year. A sensitivity analysis was carried out by removing individual studies to measure the robustness of the results. Egger and Peters tests (18) were performed to provide quantitative evidence of any publication bias (n>10).

The grading of recommendations assessment, development, and evaluation (GRADE) algorithm (19) was used to assign quality levels to the meta-analysis evidence. The overall confidence could be judged as “high,” “moderate,” “low,” or “very low.”

## RESULTS

### Literature selection

**Figure 1:**
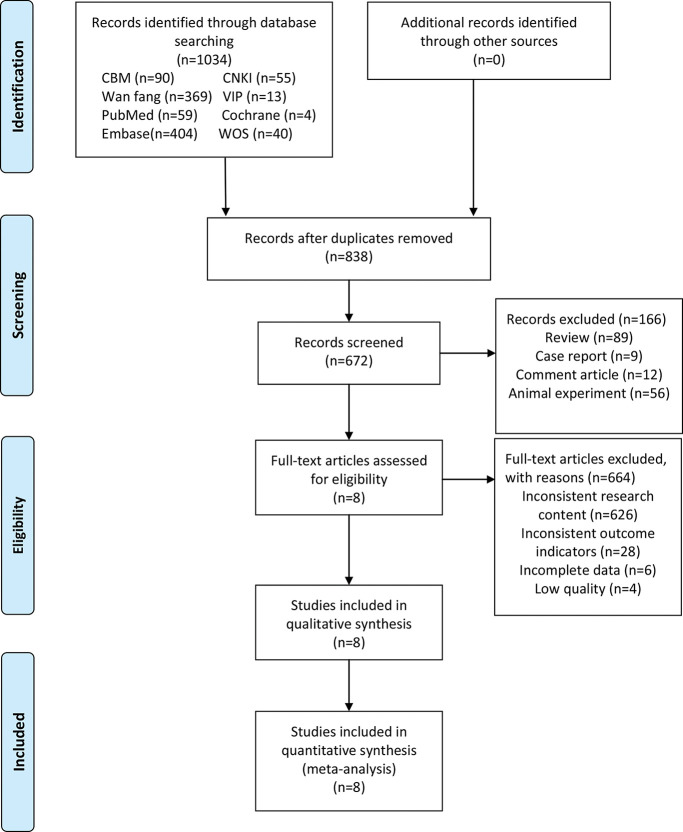
Flowchart of the search strategy and inclusion of the studies according to the preferred reporting items for systematic reviews and meta-analyses statement. CBM: Chinese Biomedical Literature Database; CNKI: China National Knowledge Infrastructure; VIP: VIP Database for Chinese Technical Periodicals; WOS: Web of Science.

A total of 1034 studies were initially identified. Of these, 196 were excluded as they were duplicate studies, and 166 were excluded following a review of the title or abstract. A total of 672 studies remained for full text review. Of these, 664 were excluded as they did not meet the eligibility criteria. Finally, the eight remaining articles (7,8,11,12,20-23) were included in this meta-analysis, including four in Chinese and four in English. All of these were case-control studies. A flowchart of the literature screening and selection process is shown in Figure 1.

### Characteristics of the studies and quality assessment

Two reviewers independently summarized the characteristics of the included studies according to the data extraction process. A total of 1669 patients were involved, which included 692 in the research group and 977 in the control group. The primary localities of the studies were distributed in three countries, six provinces, and municipalities. The median NOS score of the included studies was 6, with a range from 5 to 7, indicating that these studies were of high quality. The key characteristics of the included studies are presented in Table 2. As evaluated by the AMSTAR2 tool, this meta-analysis scored “very good.” Only questions 7 and 9 were evaluated as “No,” and the rest were evaluated as “Yes.”

### Data analysis

**Figure 2:**
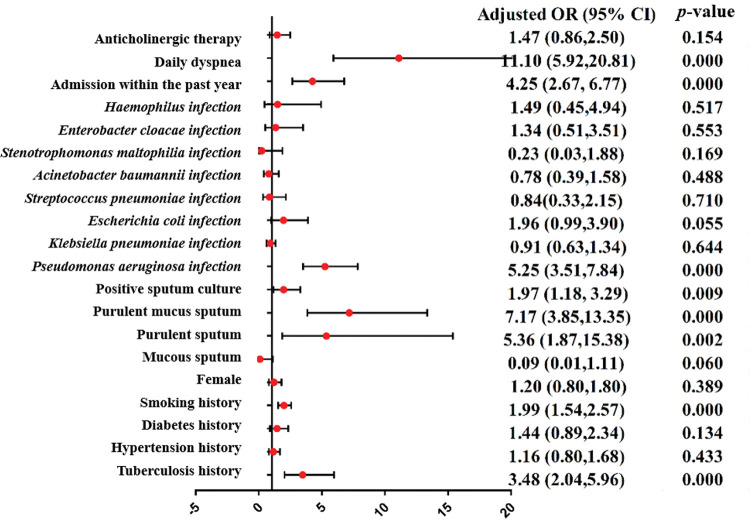
Forest-plot of the binary variable index (OR). CI, confidence interval; OR, odds ratio.

**Figure 3:**
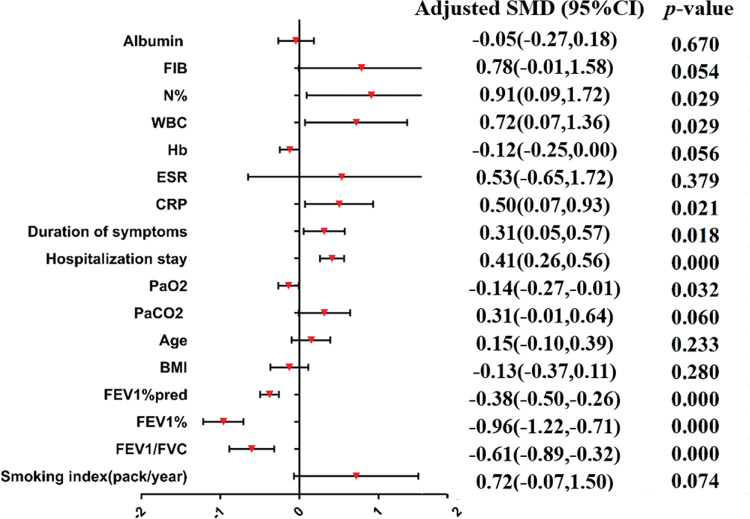
Forest-plot of the continuous variable index (SMD). BMI, body mass index; CI, confidence interval; CRP, C-reactive protein; ESR, erythrocyte sedimentation rate; FEV_1_/FVC, the ratio between forced expiratory volume in 1s and forced vital capacity; FEV_1_%, the percentage of FEV_1_;. FEV_1_%pred, FEV_1_ as a percentage of the predicted value; FIB, plasma fibrinogen; Hb, hemoglobin; N%, percentage of neutrophils; PaCO_2_, arterial carbon dioxide partial pressure; PaO_2_, arterial oxygen pressure; SMD, standardized mean difference; WBC, leukocytes.

A meta-analysis was applied to the indicators of the eight included studies. The results show that the indicators were statistically significant between the research group and control group (*p*<0.05), including tuberculosis history, smoking history, the ratio between forced expiratory volume in 1s and forced vital capacity (FEV_1_/FVC), the percentage of FEV_1_ (FEV_1_%), the FEV_1_as a percentage of the predicted value (FEV_1_%pred), purulent sputum, purulent mucus sputum, positive sputum culture, *Pseudomonas aeruginosa* infection, arterial oxygen pressure (PaO_2_), hospital stay, admission within the past year, duration of symptoms, daily dyspnea, C-reactive protein (CRP), leukocytes (WBC), and the percentage of neutrophils (N%). The results of the heterogeneity test, model, effect size, 95% CI, and *p*-values are shown in Table 3. The forest plots of the two types of variable indexes are described in Figures 2 and 3.

Reversed results of certain factors existed according to the sensitivity analysis. The lower heterogeneity and stable results emerged after excluding data on arterial carbon dioxide partial pressure (PaCO_2_) and CRP. The specific results are listed in Table 3. The results of the remaining factors were unchanged after the sensitivity analysis, suggesting that the results should be more stable.

A subgroup analysis was used to explore the sources of heterogeneity for the indicators. For the factor of age, a subgroup analysis was conducted with two groups according to the country (Asian/non-Asian). There was no change in the Asian group but statistical significance in the non-Asian group. The factor of purulent sputum was analyzed in the subgroup analysis according to the country (Asian/non-Asian). The results showed no change in the non-Asian group. In contrast, there was no statistical significance and lower heterogeneity in the Asian group. Therefore, the country where the study was conducted may be a confounding factor and source of heterogeneity, and more research will be needed in the future.

### Sensitivity analysis and GRADE evaluation

The robustness of the results in the sensitivity analysis was good, except for smoking index, body mass index (BMI), mucous sputum, purulent sputum, PaCO_2_, PaO_2_, CRP, erythrocyte sedimentation rate (ESR), hemoglobin (Hb), plasma fibrinogen (FIB), WBC, and N%. The sensitivity analysis indicated heterogeneity in the strengths of the association due to the most common biases in observational studies. The GRADE evidence of all outcomes was judged as “low” or “very low.” The results are shown in Tables 4 and 5.

## DISCUSSION

The prevention of bronchiectasis is important in the treatment of patients with COPD. However, until now, the risk factors of bronchiectasis have not been confirmed. This study demonstrated a clear relationship between patients with COPD and bronchiectasis and certain risk factors, helping us to better understand the disease. Several case-control studies included in this article suggested some risk factors for bronchiectasis in patients with COPD (Table 3). The results showed that the risk factors for bronchiectasis in COPD might include tuberculosis history, smoking history, hospitalization stay, admission within the past year, and duration of symptoms. In addition, FEV_1_/FVC, FEV_1_%, FEV_1_%pred, purulent sputum, purulent mucus sputum, positive sputum culture, *Pseudomonas aeruginosa* infection, PaO_2_, daily dyspnea, CRP, WBC, and N% were clinical symptoms of bronchiectasis. They were closely related to bronchiectasis in COPD but were not regarded as risk factors. The lung lumens and parenchyma of patients with COPD with a history of tuberculosis were destroyed, which could lead to prolonged airway inflammation duration and acceleration of lung injury and severe airflow obstruction, thus increasing the incidence of bronchiectasis (22). Therefore, patients with a history of tuberculosis should also undergo regular follow-up, although the disease has been cured. Smoking tended to affect lung function. Therefore, it is necessary for patients with COPD to quit smoking. The lung function of patients with COPD was directly impaired due to irreversible airflow limitation. The lung function indicators progressively decreased, which negatively correlated with the number of damaged lung lobes (25). This tends to induce COPD deterioration, and the second most prevalent cause of bronchiectasis was COPD (26).

Consequently, patients with COPD need to monitor lung function indicators regularly to avoid further deterioration. COPD usually has recurrent attacks and is difficult to cure. If patients cough up purulent sputum, this can lead to a considerably greater magnitude of airway dysbiosis (27). However, purulent sputum is not regarded as a risk factor for bronchiectasis in COPD. Bacterial colonization of the airway was the main inducer of airway inflammation in bronchiectasis (24). Positive sputum culture in patients with COPD demonstrated an imbalance of autoimmune function, which increased the host’s predisposition to diseases. The most common pathogenic microorganism, such as *Pseudomonas aeruginosa* (28), causes chronic inflammation and lung injury aggravations and increases the incidence of bronchiectasis. However, positive sputum culture and *Pseudomonas aeruginosa* infection were not considered risk factors for bronchiectasis in patients with COPD because these symptoms were present in bronchiectasis. The overall result of PaO_2_ described in the literature was significant, but the results were reversed after removing the studies by Pan et al. (22) or Qin (20), indicating that the robustness of the results was poor. The inconsistent results of the two studies above were likely because different blood collection times and instrument models were used in the blood gas analysis. In summary, more studies are needed to identify the relationship between PaO_2_ and bronchiectasis in patients with COPD. The chance of contact with medical staff and patients in the same hospital increased after longer hospitalization, resulting in a greater risk of nosocomial infection. The hospitalized patients were more concentrated (29); therefore, the length of hospital stay directly affects the possibility of bronchiectasis in COPD. If patients with COPD were admitted to hospital within the past year, they might have had poorer disease control and acute exacerbation. The acute exacerbation of COPD resulted in repeated injuries to the lung tissue, leading to more severe airflow obstruction, which was susceptible to bronchiectasis (30). Thus, the disease should be strictly controlled according to the medical advice given to avoid admission for acute exacerbations to reduce the possibility of bronchiectasis. A longer duration of symptoms in COPD is a critical indicator of disease deterioration. Long-term clinical symptoms relieved the patient’s resistance. The incidence of bronchiectasis was found to increase due to bronchial infection and the secretions blocking the airway (21). In conclusion, a longer duration of symptoms and hospital admissions within the past year were risk factors for bronchiectasis in COPD. The results of indicators such as purulent sputum, CRP, WBC, and N% were significant. Nonetheless, the results were reversed after removing some studies, indicating that the robustness of the results was weak. Some biases may exist in different clinical analysis instruments, and more rigorous studies are needed to identify these indexes.

In addition to the above indicators, the results including smoking index, BMI, mucous sputum, ESR, Hb, and FIB were not significant. However, the results were all reversed in the sensitivity analysis, and more clinical studies are required for supplementary verification. The smoking index may have something in common with smoking history, which tends to aggravate airway inflammation in COPD and increase the incidence of bronchiectasis. A study (31) has shown that low BMI is accompanied by a decrease in muscle mass, which may lead to depression in the strength of the respiratory muscles. Hu X et al. (32) proposed that COPD and bronchiectasis should have a high commonality in clinical symptoms, pathophysiology, and other aspects. The social burden and psychological pressure of patients were increased with the severe airway limitations related to bronchiectasis in COPD. Therefore, we should be familiar with the risk factors for bronchiectasis in patients with COPD. This will help ensure the early prevention, detection, and treatment of bronchiectasis in patients with COPD. Thus, to reduce their risk of bronchiectasis, patients with COPD should quit smoking and drinking alcohol, maintain a balanced diet, and prevent infection. We should devote equal attention to each complementary risk factor. The articles were strictly selected according to the inclusion and exclusion criteria. This study set the sources from which the authors received the diagnostic criteria for COPD and bronchiectasis. However, the final result might be affected by the interference of some factors, and there are several limitations in this meta-analysis, such as uncertainty bias in the secondary data, a limited number of articles, small total sample size, and unpredictable differences between sample sizes.

In conclusion, the presented results can be valuable to the medical community. The strengths of this review and meta-analysis include the inclusion of articles that assessed the quality of evidence evaluation using the GRADE approach. However, more studies with larger sample sizes are required. Furthermore, a multi-center case-control study is required to identify the risk factors scientifically and comprehensively for bronchiectasis in COPD. This study can be beneficial in guiding clinicians to formulate targeted prevention and treatment measures. This paper can provide recommendations for improving survival and quality of life and reducing the psychological, family, social, and medical burdens of patients with COPD and clinical guidance for reducing the incidence of bronchiectasis in patients with COPD.

## AUTHOR CONTRIBUTIONS

Zhang XX was responsible for the topic selection and manuscript drafting. Zhang XX and Zhang HY contributed to the data acquisition and analysis. All authors contributed to the data interpretation and critical revisions of the manuscript. Pang LJ and Lv XD were responsible for the final decisions on data extraction and the quality assessment. Lv XD was responsible for funding and controlling the project.

## Figures and Tables

**Table 1:** Literature search strategy of the PubMed database

#1	“Pulmonary Disease, Chronic Obstructive” [Mesh]
#2	COPD
#3	Chronic Obstructive Pulmonary Disease
#4	COAD
#5	Chronic Obstructive Airway Disease
#6	Chronic Obstructive Lung Disease
#7	Airflow Obstruction, Chronic
#8	Airflow Obstructions, Chronic
#9	Chronic Airflow Obstructions
#10	Chronic Airflow Obstruction
#11	#1 OR #2 OR #3 OR #4 OR #5 OR #6 OR #7 OR #8 OR #9 OR #10
#12	“Bronchiectasis” [Mesh]
#13	Bronchiectases
#14	#12 OR #13
#15	“Risk Factors” [Mesh]
#16	Factor, Risk
#17	Factors, Risk
#18	Risk Factor
#19	Population at Risk
#20	Risk, Population at
#21	Populations at Risk
#22	Risk, Populations at
#23	#15 OR #16 OR #17 OR #18 OR #19 OR #20 OR #21 OR #22
#24	#11 AND #14 AND #23

COAD, Chronic Obstructive Airways Disease; COPD, Chronic obstructive pulmonary disease.

**Table 2:** Summary of the study design and study characteristics.

Study	Publication year	Locality	Sample size (T/C)	Quality assessment (NOS score)	Research factors	Age (T/C)	Sex (Male/Female)	Diagnostic criteria	Funder
Qin (20)	2018	Jilin	198/282	6	1.4.6.8.9.10.11.16.17.18.19.20.22.24.25.26.30.31.32.33.34.35	69.88±9.72/ 69.95±9.73	257/223	b (2017 version) HRCT	NA
Liu et al. (21)	2019	Anhui	57/96	5	1.2.3.4.10.11.13.16.17.18.24.25.26.28.30.31.32.33.34	69.57±9.64/ 66.34±9.52	94/59	b HRCT	Anhui Provincial Health Department (13ZC024)
Pan et al. (22)	2019	Shanghai	135/217	6	1.2.3.4.6.8.9.10.11.16.17.18.19.20.22.24.25.31.32.33.34.35	62.81±10.42/ 64.37±9.15	184/168	a (2013 revision) HRCT	NA
Zhao (23)	2015	Hebei	86/114	5	5.6.7.10.11.12.13.14.15.16.17.18.19.21.27.29	67.79±9.27/ 69.48±10.02	173/27	a (2007 revision) b (2011 version) HRCT	NA
Jin et al. (7)	2016	Beijing	87/103	6	1.4.5.8.9.10.11.30.35.36.37	77.0/78.9	121/69	clinical diagnosis HRCT	National Natural Science Foundation of China (81170039, 81470239); Beijing Talent Training Foundation (No. 2009D003003000002)
Martínez-García et al. (8)	2011	Spain	53/39	7	1.4.5.6.10.11.12.13.16.28.37	72.6/69.1	91/1	clinical diagnosis HRCT	A public grant from the Sociedad Valenciana de Neumología
Yu et al. (12)	2019	Tianjin	43/90	6	1.5.6.8.10.11.16.17.20.21.23.26.27.30.33.35.36	71.02±8.47/ 69.78±8.24	83/50	clinical diagnosis HRCT	None
Arram et al. (11)	2012	Egypt	33/36	7	5.6.7.10.11.12.13.14.15.16.17.19.23.27.29	63.79±5.41/ 56.50±5.56	4/65	b (2009 version) HRCT	NA

1. Tuberculosis history 2. Hypertension history 3. Diabetes history 4. Smoking history 5. Smoking index (pack/year) 6. FEV_1_/FVC 7. FEV_1_% 8. FEV_1_%pred 9. BMI 10. Age 11. Female 12. Mucous sputum 13. Purulent sputum 14. Purulent mucus sputum, 15. Positive sputum culture, 16. *Pseudomonas aeruginosa* infection 17. *Klebsiella pneumoniae* infection, 18. *Escherichia coli* infection 19. *Streptococcus pneumoniae* infection 20. *Acinetobacter baumannii* infection 21. *Stenotrophomonas maltophilia* infection 22. *Enterobacter cloacae* infection 23. *Haemophilus* infection, 24. PaCO_2_ 25. PaO_2_ 26. Hospital stays 27. Admission within the past year 28. Duration of symptoms 29. Daily dyspnea 30. CRP 31. ESR 32. Hb 33. WBC 34. N% 35. FIB 36. Albumin 37. Anticholinergic therapy.

a. Guidelines for the diagnosis and treatment of chronic obstructive pulmonary disease.

b. The Global Initiative for Chronic Obstructive Lung Disease.

BMI, body mass index; C, control group; CRP, C-reactive protein; ESR, erythrocyte sedimentation rate; FEV_1_/FVC, the ratio between forced expiratory volume in 1s and forced vital capacity; FEV_1_%, the percentage of FEV_1_;. FEV_1_%pred, FEV_1_ as a percentage of the predicted value; FIB, plasma fibrinogen; Hb, hemoglobin; HRCT, High Resolution Computed Tomography; N%, percentage of neutrophils; NA, unclear or not mentioned; None, no funding; PaCO_2_, arterial carbon dioxide partial pressure; PaO_2_, arterial oxygen pressure; WBC, leukocyte; T, trial group.

**Table 3:** Results of the meta-analysis of various indicators.

		Heterogeneity test						Sensitivity analysis		
Factors	Number	Q-test	I^2^ (%)	Effect model	Effect selection	Effect size and 95%CI	*p-*value	I^2^ (%)	*p*-value	excluded
Tuberculosis history	6	0.023	61.7%	Random	OR	3.48 (2.04,5.96)	0.000			
Hypertension history	2	0.844	0.0%	Fixed	OR	1.16 (0.80,1.68)	0.433			
Diabetes history	2	0.243	26.7%	Fixed	OR	1.44 (0.89,2.34)	0.134			
Smoking history	5	0.141	42.0%	Fixed	OR	1.99 (1.54,2.57)	0.000			
Smoking index (pack/year)	5	0.000	95.5%	Random	SMD	0.72 (-0.07,1.50)	0.074	95.0%	0.025	Yu et al. (12)
FEV_1_/FVC	6	0.000	81.7%	Random	SMD	-0.61 (-0.89,-0.32)	0.000			
FEV_1_%	2	0.198	39.7%	Fixed	SMD	-0.96 (-1.22,-0.71)	0.000			
FEV1%pred	4	0.977	0.0%	Fixed	SMD	-0.38 (-0.50,-0.26)	0.000			
BMI	3	0.031	71.1%	Random	SMD	-0.13 (-0.37,0.11)	0.280	0.0%	0.003	Pan et al. (22)
Age	8	0.000	81.7%	Random	SMD	0.15 (-0.10,0.39)	0.233			
Female sex	8	0.017	59.0%	Random	OR	1.20 (0.80,1.80)	0.389			
Mucous sputum	3	0.000	90.9%	Random	OR	0.09 (0.01,1.11)	0.060	82.4%	0.030	Martínez-García et al. (8)
Purulent sputum	4	0.039	64.1%	Random	OR	5.36 (1.87,15.38)	0.002	68.4% 72.6%	0.120 0.073	Liu et al. (21) / Zhao (23)
Purulent mucus sputum	2	0.751	0.0%	Fixed	OR	7.17 (3.85,13.35)	0.000			
Positive sputum culture	2	0.638	0.0%	Fixed	OR	1.97 (1.18, 3.29)	0.009			
*Pseudomonas aeruginosa* infection	7	0.916	0.0%	Fixed	OR	5.25 (3.51,7.84)	0.000			
*Klebsiella pneumoniae* infection	6	0.096	46.6%	Fixed	OR	0.91 (0.63,1.34)	0.644			
*Escherichia coli* infection	4	0.747	0.0%	Fixed	OR	1.96 (0.99,3.90)	0.055			
*Streptococcus pneumoniae* infection	4	0.860	0.0%	Fixed	OR	0.84 (0.33,2.15)	0.710			
*Acinetobacter baumannii* infection	3	0.687	0.0%	Fixed	OR	0.78 (0.39,1.58)	0.488			
*Stenotrophomonas maltophilia* infection	2	0.836	0.0%	Fixed	OR	0.23 (0.03,1.88)	0.169			
*Enterobacter cloacae* infection	2	0.865	0.0%	Fixed	OR	1.34 (0.51,3.51)	0.553			
*Haemophilus* infection	2	0.324	0.0%	Fixed	OR	1.49 (0.45,4.94)	0.517			
PaCO_2_	3	0.003	82.5%	Random	SMD	0.31 (-0.01,0.64)	0.060	0.0%	0.064	Liu et al. (21)
PaO_2_	3	0.518	0.0%	Fixed	SMD	-0.14 (-0.27,-0.01)	0.032	23.9% 0.0%	0.085 0.326	Pan et al. (22) / Qin (20)
Hospital stay	3	0.749	0.0%	Fixed	SMD	0.41 (0.26,0.56)	0.000			
Admission within the past year	3	0.250	27.9%	Fixed	OR	4.25 (2.67, 6.77)	0.000			
Duration of symptoms	2	0.554	0.0%	Fixed	SMD	0.31 (0.05,0.57)	0.018			
Daily dyspnea	2	0.343	0.0%	Fixed	OR	11.10 (5.92,20.81)	0.000			
CRP	4	0.000	89.0%	Random	SMD	0.50 (0.07,0.93)	0.021	91.1% 0.0%	0.060 0.001	Jin et al. (7) / Qin (20)
ESR	3	0.000	98.5%	Random	SMD	0.53 (-0.65,1.72)	0.379	0.0%	0.000	Liu (21)
Hb	3	0.138	49.5%	Fixed	SMD	-0.12 (-0.25,0.00)	0.056	0.0%	0.007	Qin (20)
WBC	4	0.000	95.7%	Random	SMD	0.72 (0.07,1.36)	0.029	0.0%	0.294	Qin (20) / Pan et al. (22)
N%	3	0.000	96.9%	Random	SMD	0.91 (0.09,1.72)	0.029	95.9% 98.4%	0.280 0.294	Qin (20) / Pan et al. (22)
FIB	4	0.000	97.3%	Random	SMD	0.78 (-0.01,1.58)	0.054	80.3% 97.6%	0.025 0.030	Qin (20) / Yu et al. (12)
Albumin	2	0.320	0.0%	Fixed	SMD	-0.05 (-0.27,0.18)	0.670			
Anticholinergic therapy	2	0.536	0.0%	Fixed	OR	1.47 (0.86,2.50)	0.154			

The blank lines in the sensitivity analysis columns indicate that the results were stable.

BMI, body mass index; CI, confidence interval; CRP, C-reactive protein; ESR, erythrocyte sedimentation rate; FEV_1_/FVC, the ratio between forced expiratory volume in 1 s and forced vital capacity; FEV_1_%, the percentage of FEV_1_;. FEV_1_%pred, FEV_1_ as a percentage of the predicted value; FIB, plasma fibrinogen; GRADE, grading of recommendations assessment, development, and evaluation; Hb, hemoglobin; N%, percentage of neutrophils; OR, odds ratio; PaCO_2_, arterial carbon dioxide partial pressure; PaO_2_, arterial oxygen pressure; SMD, standardized mean difference; WBC, leukocytes

**Table 4:** GRADE evidence profile.

Quality assessment							Summary of findings				
							Number of patients		Effect		
No. of studies	Study design	Risk of bias	Inconsistency	Indirectness	Imprecision	Publication bias	Bronchiectasis	No bronchiectasis	Relative (95%CI)	Absolute (95%CI)	Quality
Tuberculosis history											
6	Observational study	not serious	serious^a^	not serious	serious^o^	not found	176/573	103/827	RR 2.49 (1.65 to 3.75)	96 per 1000	⊕○○○
Smoking history											
5	Observational study	not serious	not serious	not serious	not serious	not found	367/530	393/737	RR 1.25 (1.15 to 1.35)	635 per 1000	⊕⊕○○
*Pseudomonas aeruginosa* infection											
7	Observational study	not serious	not serious	not serious	serious^o^	not found	107/605	35/874	RR 4.36 (3.04 to 6.27)	28 per 1000	⊕○○○
Purulent sputum											
4	Observational study	not serious	serious^b^	not serious	serious^o^	not found	83/229	35/285	RR 3.09 (1.68 to 5.69)	70 per 1000	⊕○○○
Purulent mucus sputum											
2	Observational study	not serious	not serious	not serious	serious^ o^	not found	57/119	17/150	RR 4.18 (2.57 to 6.79)	122 per 1000	⊕○○○
Positive sputum culture											
2	Observational study	not serious	not serious	not serious	serious^o^	not found	51/119	41/150	RR 1.56 (1.11 to 2.17)	284 per 1000	⊕○○○
Admission within the past year											
3	Observational study	not serious	not serious	not serious	serious^o^	not found	110/162	85/240	RR 1.82 (1.51 to 2.20)	222 per 1000	⊕○○○
Daily dyspnea											
2	Observational study	not serious	not serious	not serious	serious^o^	not found	101/119	54/150	RR 2.40 (1.91 to 3.01)	294 per 1000	⊕○○○
Hypertension history											
2	Observational study	not serious	not serious	not serious	serious^o,p^	not found	76/192	113/313	RR 1.10 (0.87 to 1.38)	362 per 1000	⊕○○○
Diabetes history											
2	Observational study	not serious	not serious	not serious	serious^o,p^	not found	40/192	50/313	RR 1.32 (0.92 to 1.90)	208 per 1000	⊕○○○
Female											
8	Observational study	not serious	serious^c^	not serious	serious^ p^	not found	259/692	374/977	RR 1.09 (0.91 to 1.31)	328 per 1000	⊕○○○
Mucous sputum											
3	Observational study	not serious	serious^d^	not serious	serious^o,p^	not found	69/172	156/189	RR 0.29 (0.05 to 1.66)	546 per 1000	⊕○○○
*Klebsiella pneumoniae* infection											
6	Observational study	not serious	not serious	not serious	serious^o,p^	not found	47/552	78/835	RR 0.92 (0.66 to 1.30)	49 per 1000	⊕○○○
*Escherichia coli* infection											
4	Observational study	not serious	not serious	not serious	serious^o,p^	not found	19/476	15/709	RR 1.92 (0.99 to 3.75)	21 per 1000	⊕○○○
*Streptococcus pneumoniae* infection											
4	Observational study	not serious	not serious	not serious	serious^o,p^	not found	7/452	11/649	RR 0.84 (0.35 to 2.05)	10 per 1000	⊕○○○
*Acinetobacter baumannii* infection											
3	Observational study	not serious	not serious	not serious	serious^o,p^	not found	12/376	24/589	RR 0.79 (0.40 to 1.55)	41 per 1000	⊕○○○
*Stenotrophomonas maltophilia* infection											
2	Observational study	not serious	not serious	not serious	serious^o,p^	not found	0/129	6/204	RR 0.23 (0.03 to 1.86)	30 per 1000	⊕○○○
*Enterobacter cloacae* infection											
2	Observational study	not serious	not serious	not serious	serious^o,p^	not found	8/333	9/499	RR 1.33 (0.52 to 3.42)	18 per 1000	⊕○○○
*Haemophilu*s infection											
2	Observational study	not serious	not serious	not serious	serious^o,p^	not found	6/76	5/126	RR 1.42 (0.50 to 3.98)	69 per 1000	⊕○○○
Anticholinergic therapy											
2	Observational study	not serious	not serious	not serious	serious^o,p^	not found	107/140	96/142	RR 1.11 (0.96 to 1.28)	713 per 1000	⊕○○○
FEV_1_/FVC											
6	Observational study	not serious	serious^e^	not serious	not serious	not found	548	778	-	SMD-0.61 (-0.89 to -0.32)	⊕○○○
FEV_1_%											
2	Observational study	not serious	not serious	not serious	serious^q^	not found	119	150	-	SMD-0.96 (-1.22 to -0.71)	⊕○○○
FEV_1_%pred											
4	Observational study	not serious	not serious	not serious	not serious	not found	463	692	-	SMD-0.38 (-0.50 to -0.26)	⊕⊕○○
PaO_2_											
3	Observational study	not serious	not serious	not serious	not serious	not found	390	595	-	SMD-0.14 (-0.27 to -0.01)	⊕⊕○○
Hospital stay											
3	Observational study	not serious	not serious	not serious	not serious	not found	298	468	-	SMD 0.41 (0.26 to 0.56)	⊕⊕○○
Duration of symptoms											
2	Observational study	not serious	not serious	not serious	serious^q^	not found	110	135	-	SMD 0.31 (0.05 to 0.57)	⊕○○○
CRP											
4	Observational study	not serious	serious^ f^	not serious	not serious	not found	385	571	-	SMD 0.50 (0.07 to 0.93)	⊕○○○
WBC											
4	Observational study	not serious	serious^g^	not serious	not serious	not found	433	685	-	SMD 0.72 (0.07 to 1.36)	⊕○○○
N%											
3	Observational study	not serious	serious^h^	not serious	not serious	not found	816	1116	-	SMD 0.91 (0.09 to 1.72)	⊕○○○
Smoking index (pack/year)											
5	Observational study	not serious	serious^i^	not serious	not serious	not found	302	382	-	SMD 0.72 (-0.07 to 1.50)	⊕○○○
BMI											
3	Observational study	not serious	serious^ j^	not serious	not serious	not found	420	602	-	SMD -0.13 (-0.37 to 0.11)	⊕○○○
Age											
8	Observational study	not serious	serious^k^	not serious	not serious	not found	692	977	-	SMD 0.15 (-0.10 to 0.39)	⊕○○○
PaCO_2_											
3	Observational study	not serious	serious^l^	not serious	not serious	not found	390	595	-	SMD 0.31 (-0.01 to 0.64)	⊕○○○
ESR											
3	Observational study	not serious	serious^m^	not serious	not serious	not found	390	595	-	SMD 0.53 (-0.65 to 1.72)	⊕○○○
Hb											
3	Observational study	not serious	not serious	not serious	not serious	not found	390	595	-	SMD -0.12 (-0.25 to 0.00)	⊕⊕○○
FIB											
4	Observational study	not serious	serious^n^	not serious	not serious	not found	463	692	-	SMD 0.78 (-0.01 to 1.58)	⊕○○○
Albumin											
2	Observational study	not serious	not serious	not serious	serious^q^	not found	130	193	-	SMD -0.05 (-0.27 to 0.18)	⊕○○○

BMI, body mass index; CI, credible interval; CRP, C-reactive protein; ESR, erythrocyte sedimentation rate; FEV_1_/FVC, the ratio between forced expiratory volume in 1s and forced vital capacity; FEV_1_%, the percentage of FEV_1_;. FEV_1_%pred, FEV_1_ as a percentage of the predicted value; FIB, plasma fibrinogen; GRADE, grading of recommendations assessment, development, and evaluation; Hb, hemoglobin; N%, percentage of neutrophils; PaCO_2_, arterial carbon dioxide partial pressure; PaO_2_, arterial oxygen pressure; RR, risk ratio; SMD, standardized mean difference; WBC, leukocytes.

GRADE Working Group grades of evidence High quality(⊕⊕⊕⊕): Further research is unlikely to change our confidence in the estimate of effect. Moderate quality(⊕⊕⊕○): Further research is likely to have an important impact on our confidence in the estimate of effect and may change the estimate. Low quality(⊕⊕○○): Further research is very likely to have an important impact on our confidence in the estimate of effect and is likely to change the estimate. Very low quality(⊕○○○): We are very uncertain about the estimate.

a. I^2^=61.7%; b. I^2^=64.1%; c. I^2^=59.0%; d. I^2^=90.9%; e. I^2^=81.7%; f. I^2^=89.0%; g. I^2^=95.7%; h. I^2^=96.9%; i. I^2^=95.5%; j. I^2^=71.1%; k. I^2^=81.7%; l. I^2^=82.5%; m. I^2^=98.5%; n. I^2^=97.3%; o. The total sample size was less than the optimal information size, p. The 95%CI of the pooled estimate included one or no effect; q. The total sample size was less than 400.

**Table 5:** GRADE summary of findings.

Outcome	Anticipated absolute effects (95%CI)		Relative effect (95%CI)	No. of participants (Studies)	Quality	Comments
	Risk with no bronchiectasis	Risk with bronchiectasis				
Tuberculosis history	96 per 1000	239 per 1000 (158 to 360)	RR 2.49 (1.65 to 3.75)	1400 (6)	⊕○○○	
Smoking history	635 per 1000	794 per 1000 (730 to 857)	RR 1.25 (1.15 to 1.35)	1267 (5)	⊕⊕○○	
*Pseudomonas aeruginosa* infection	28 per 1000	122 per 1000 (85 to 176)	RR 4.36 (3.04 to 6.27)	1479 (7)	⊕○○○	
Purulent sputum	70 per 1000	216 per 1000 (117 to 398)	RR 3.09 (1.68 to 5.69)	514 (4)	⊕○○○	
Purulent mucus sputum	122 per 1000	510 per 1000 (314 to 828)	RR 4.18 (2.57 to 6.79)	269 (2)	⊕○○○	
Positive sputum culture	284 per 1000	443 per 1000 (315 to 616)	RR 1.56 (1.11 to 2.17)	269 (2)	⊕○○○	
Admission within the past year	222 per 1000	404 per 1000 (335 to 488)	RR 1.82 (1.51 to 2.20)	402 (3)	⊕○○○	
Daily dyspnea	294 per 1000	706 per 1000 (562 to 885)	RR 2.40 (1.91 to 3.01)	269 (2)	⊕○○○	
Hypertension history	362 per 1000	398 per 1000 (315 to 500)	RR 1.10 (0.87 to 1.38)	505 (2)	⊕○○○	
Diabetes history	208 per 1000	275 per 1000 (191 to 395)	RR 1.32 (0.92 to 1.90)	505 (2)	⊕○○○	
Female	328 per 1000	358 per 1000 (298 to 430)	RR 1.09 (0.91 to 1.31)	1669 (8)	⊕○○○	
Mucous sputum	546 per 1000	158 per 1000 (27 to 906)	RR 0.29 (0.05 to 1.66)	361 (3)	⊕○○○	
*Klebsiella pneumoniae* infection	49 per 1000	45 per 1000 (32 to 64)	RR 0.92 (0.66 to 1.30)	1387 (6)	⊕○○○	
*Escherichia coli* infection	21 per 1000	40 per 1000 (20 to 79)	RR 1.92 (0.99 to 3.75)	1185 (4)	⊕○○○	
*Streptococcus pneumoniae* infection	10 per 1000	8 per 1000 (4 to 21)	RR 0.84 (0.35 to 2.05)	1101 (4)	⊕○○○	
*Acinetobacter baumannii* infection	41 per 1000	32 per 1000 (16 to 64)	RR 0.79 (0.40 to 1.55)	965 (3)	⊕○○○	
*Stenotrophomonas maltophilia* infection	30 per 1000	7 per 1000 (1 to 56)	RR 0.23 (0.03 to 1.86)	333 (2)	⊕○○○	
*Enterobacter cloacae* infection	18 per 1000	24 per 1000 (9 to 62)	RR 1.33 (0.52 to 3.42)	832 (2)	⊕○○○	
*Haemophilus* infection	69 per 1000	98 per 1000 (35 to 275)	RR 1.42 (0.50 to 3.98)	202 (2)	⊕○○○	
Anticholinergic therapy	713 per 1000	791 per 1000 (684 to 913)	RR 1.11 (0.96 to 1.28)	282 (2)	⊕○○○	
FEV_1_/FVC	The mean FEV_1_/FVC in the control group was 0	The mean FEV_1_/FVC in the trial group was 0.61 standard deviations lower (0.89 lower to 0.32 lower)	-	1326 (6)	⊕○○○	
FEV_1_%	The mean FEV_1_% in the control group was 0	The mean FEV_1_% in the trial group was 0.96 standard deviations lower (1.22 lower to 0.71 lower)	-	269 (2)	⊕○○○	
FEV_1_%pred	The mean FEV_1_%pred in the control group was 0	The mean FEV_1_%pred in the trial group was 0.38 standard deviations lower (0.50 lower to 0.26 lower)	-	1155 (4)	⊕⊕○○	
PaO_2_	The mean PaO_2_ in the control group was 0	The mean PaO_2_ in the trial group was 0.14 standard deviations lower (0.27 lower to 0.01 lower)	-	985 (3)	⊕⊕○○	
Hospital stay	The mean hospital stay in the control group was 0	The mean hospital stay in the trial group was 0.41 standard deviations higher (0.26 lower to 0.56 higher)	-	766 (3)	⊕⊕○○	
Duration of symptoms	The mean duration of symptoms in the control group was 0	The mean duration of symptoms in the trial group was 0.31 standard deviations higher (0.05 lower to 0.57 higher)	-	245 (2)	⊕○○○	
CRP	The mean CRP in the control group was 0	The mean CRP in the trial group was 0.50 standard deviations higher (0.07 lower to 0.93 higher)	-	956 (4)	⊕○○○	
WBC	The mean WBC in the control group was 0	The mean WBC in the trial group was 0.72 standard deviations higher (0.07 lower to 1.36 higher)	-	1118 (4)	⊕○○○	
N%	The mean N% in the control group was 0	The mean N% in the trial group was 0.91 standard deviations higher (0.09 lower to 1.72 higher)	-	1932 (3)	⊕○○○	
Smoking index (pack/year)	The mean smoking index in the control group was 0	The mean smoking index in the trial group was 0.72 standard deviations higher (0.07 lower to 1.50 higher)	-	684 (5)	⊕○○○	
BMI	The mean BMI in the control group was 0	The mean BMI in the trial group was 0.13 standard deviations lower (0.37 lower to 0.11 higher)	-	1022 (3)	⊕○○○	
Age	The mean age in the control group was 0	The mean age in the trial group was 0.15 standard deviations higher (0.10 lower to 0.39 higher)	-	1669 (8)	⊕○○○	
PaCO_2_	The mean PaCO_2_ in the control group was 0	The mean PaCO_2_ in the trial group was 0.31 standard deviations higher (0.01 lower to 0.64 higher)	-	985 (3)	⊕○○○	
ESR	The mean ESR in the control group was 0	The mean ESR in the trial group was 0.53 standard deviations higher (0.65 lower to 1.72 higher)	-	985 (3)	⊕○○○	
Hb	The mean Hb in the control group was 0	The mean Hb in the trial group was 0.12 standard deviations lower (0.25 lower to 0.00 higher)	-	985 (3)	⊕⊕○○	
FIB	The mean FIB in the control group was 0	The mean FIB in the trial group was 0.78 standard deviations higher (0.01 lower to 1.58 higher)	-	1155 (4)	⊕○○○	
Albumin	The mean albumin in the control group was 0	The mean albumin in the trial group was 0.05 standard deviations lower (0.27 lower to 0.18 higher)	-	323 (2)	⊕○○○	

BMI, body mass index; CI, confidence interval; CRP, C-reactive protein; ESR, erythrocyte sedimentation rate; FEV_1_/FVC, the ratio between forced expiratory volume in 1s and forced vital capacity; FEV_1_%, the percentage of FEV_1_;. FEV_1_%pred, FEV_1_ as a percentage of the predicted value; FIB, plasma fibrinogen; GRADE, grading of recommendations assessment, development, and evaluation; Hb, hemoglobin; N%, percentage of neutrophils; PaCO_2_, arterial carbon dioxide partial pressure; PaO_2_, arterial oxygen pressure; RR, risk ratio; WBC, leukocytes.
